# A Novel Multi-Angle SAR Imaging System and Method Based on an Ultrahigh Speed Platform

**DOI:** 10.3390/s19071701

**Published:** 2019-04-10

**Authors:** Wensheng Chang, Haihong Tao, Guangcai Sun, Yuqi Wang, Zheng Bao

**Affiliations:** National Laboratory of Radar Signal Processing, Xidian University, Xi’an 710071, China; victorycws@163.com (W.C.); hhtao@xidian.edu.cn (H.T.); xdwangyuqi@163.com (Y.W.); zhbao@mail.xidian.edu.cn (Z.B.)

**Keywords:** SAR imaging, multi-angle SAR, improved RMA, SAR image fusion

## Abstract

Considering the difficulty of pulse repetition frequency (PRF) design in multi-angle SAR when using ultra-high speed platforms, a multi-angle SAR imaging system in a unified coordinate system is proposed. The digital multi-beamforming is used in the system and multi-angle SAR data can be obtained in one flight. Therefore, the system improves the efficiency of data recording. An improved range migration algorithm (RMA) is used for data processing, and imaging is made in a unified imaging coordinate system. The resolution of different view images is the same, and there is a fixed delay between the images. On this basis, the SAR image fusion is performed after image matching. The results of simulation and measured data confirm the effectiveness of the system and the method.

## 1. Introduction

Synthetic Aperture Radar (SAR) imaging is able to work day and night under all weather conditions [1]. Therefore, it has wide applications in topographic mapping, environmental monitoring and information acquisition, but the electromagnetic scattering property of a complex object varies with incidence angle [2]. In order to meet requirements of omnidirectional observation, it is necessary to implement new research on SAR imaging systems. The multi-angle SAR imaging system has attracted considerable attention [2,3,4,5,6,7,8].

The electromagnetic scattering property varies with incidence, so the SAR imaging is greatly affected by the incidence angle [3]. When the target is observed from one angle, since it is occluded, or the scattering coefficient of the angle is low, the complete information of the target cannot be obtained, but multi-angle SAR observes the target from different angle, and it can obtain as much information as possible about the target. The current multi-angle SAR includes spotlight SAR [4], wide azimuth beam SAR [5] and multiple flight paths SAR [6] In the spotlight SAR, the antenna is steered to increase extend the synthetic time and to observe targets from different angles. In this mode, the azimuth bandwidth of the signal may greater than the PRF, which causes spectrum ambiguity and makes signal processing more complicated [4]. The spotlight SAR expands observation angle but reduces the imaging scope. When using an ultra-high speed platform, the azimuth bandwidth of the signal becomes large, and a very large PRF is required. The wide beam angle SAR increases the beam width and obtains echoes of targets from different angles. The wide beam SAR increase the imaging scope, but the two-dimensional spectrum is a sector. This means increased range cell migration (RCM) and severe coupling of range and azimuth [5]. The error of range cell migration compensation in the frequency domain will affect the imaging accuracy. The back projection algorithm can completely compensate the RCM in time domain, but it needs a lot of calculations [7]. Multi angle observation can be realized by multiple flight paths [6], and a large imaging scope can be obtained, but the flight efficiency is low and the cost is high.

For the above problems, a new multi-angle SAR imaging system is proposed in this paper. Digital multi-beamforming is used to obtain SAR data from different angles. The digital T/R modules are divided into three groups and three sets of receiving feeders are used to obtain the multi-beam signals in the time domain. On this basis, an improved RMA in a unified coordinate system is proposed. Modified Stolt interpolation was proposed to correct the distorted spectrum in squinted SAR and improve the efficiency of the spectrum. Then, imaging is performed in a uniform coordinate system. The images of different views only have a translational relationship in azimuth, which can achieve fast matching of multi-angle images. This multi-angle SAR is not required to adjust the antenna direction, nor large beam angle, which reduces the equipment requirements. SAR data from different angles can be obtained in one flight, which reduces experimental costs.

## 2. A Multi-angle SAR Imaging System and Signal Model on a High-Speed Platform

**Figure 1:**
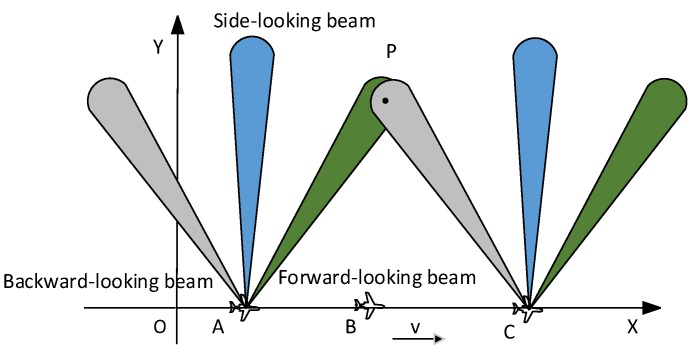
Model of multi-angle SAR imaging system.

As shown in Figure 1, the multi-angle SAR imaging system proposed in this paper adopts the digital multi-beamforming, which uses the same antenna to form multiple beams. The squint angles of three beams are different. The date of forward-looking beam, side-looking beam and backward-looking beam are recorded simultaneously, and the data received by each channel are independent from each other.

### 2.1. Digital Multi-Beamforming

**Figure 2:**
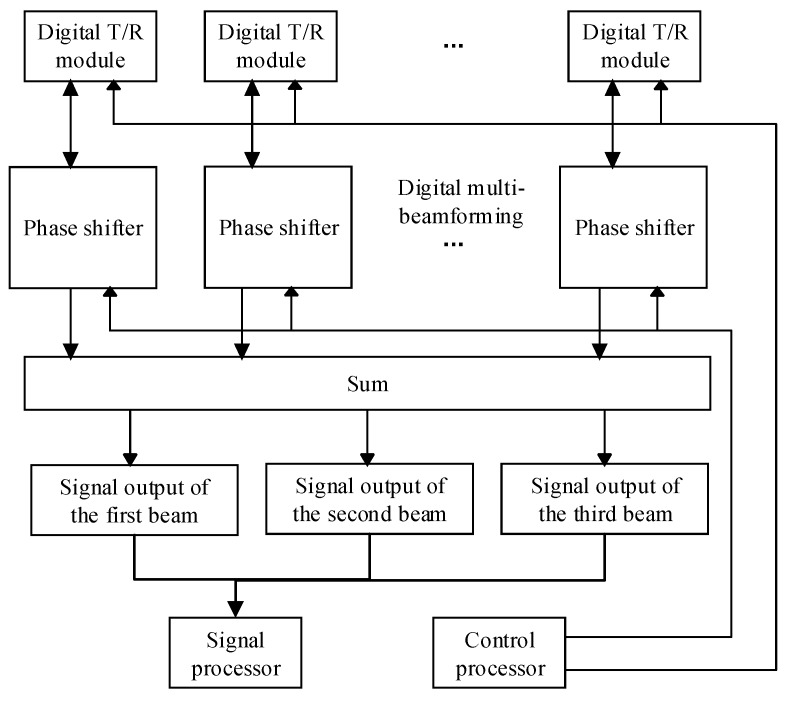
Reception of digital multi-beamforming.

There are two ways to obtain multi-beam data. One way is using one set of receiving feeders to separate multi-beam data in the Doppler domain, the other way is using multiple sets of feeders to obtain multi-beam data in time domain. When multi-beam data is separated in the Doppler domain, the Doppler bandwidth of the multi-beam signal is large. To prevent spectrum aliasing, a large PRF is required. Reference [8] gives a design to reduce the PRF, and the PRF is the sum of the Doppler bandwidth of multi-beam signals, but the method limits beam pointing. In addition, when using the ultrahigh speed platform, the Doppler bandwidth of the multi-beam signal becomes large. As a result, a large PRF is required. As shown in Figure 2, the multi-angle SAR imaging system proposed in this paper uses three sets of receiving feeders and the digital T/R modules are divided into three groups, each with independent receiving feeder and phase shifter. The multi-beam data are separated in the time domain. Thus, the PRF is equal to the Doppler bandwidth of a single beam. At the same time, there is no restriction on the direction of the beam, and the required beam pointing can be set. When the scattering angles vary from 20° to −20°, the results of the imaging will be different [3]. In order to get as much information as possible in the scene, the difference in the direction of the three beams is at least 20°.

### 2.2. Signal Model

**Figure 3:**
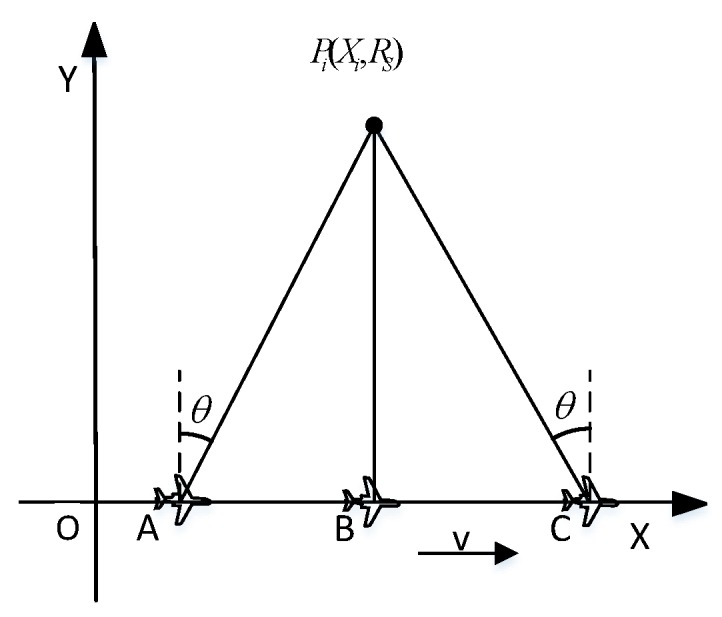
Multi-angle SAR signal model.

As shown in Figure 3, the speed of the carrier is v and the wavelength is λ. Taking the three beams as an example, in data collecting, the echo data of target $P_{i}\left(X_{i},R_{s}\right)$ from forward-looking beam is firstly obtained. When the carrier is located at A, the forward-looking beam center points to the target $P_{i}$. At this time, the squint angle is θ, and the beam-width of forward-looking beam is $\theta_{BW1}$. When $\theta_{BW1}$ is small, the Doppler bandwidth is approximately

(1) $$BW1=\frac{2v}{\lambda }\left[\sin(\theta +\frac{\theta_{BW1}}{2})-\sin(\theta -\frac{\theta_{BW1}}{2})\right]\approx \frac{2v}{\lambda }\cos\theta \cdot \theta_{BW1}$$

Then, the echo data of target $P_{i}$ from a side-looking beam is obtained. When the aircraft is at *B*, the center of the side-looking beam points to the target $P_{i}$, the beam-width of side-looking beam is $\theta_{BW2}$, and the Doppler bandwidth of side-looking beam is

(2) $$BW2=\frac{2v}{\lambda }\left[\sin(\frac{\theta_{BW2}}{2})-\sin(\frac{\theta_{BW2}}{2})\right]\approx \frac{2v}{\lambda }\theta_{BW2}$$

Finally, the echo data of target $P_{i}$ from backward -looking beam is obtained. When the aircraft is at *C*, the center of the backward-looking beam points to the target $P_{i}$, the backward-looking beam-width is $\theta_{BW3}$, and the Doppler bandwidth of backward-looking beam is

(3) $$BW3=\frac{2v}{\lambda }\left[\sin(-\theta +\frac{\theta_{BW3}}{2})-\sin(-\theta -\frac{\theta_{BW3}}{2})\right]\approx \frac{2v}{\lambda }\cos\theta \cdot \theta_{BW3}$$

The beam-width of the phased array antenna is $\theta_{BW}=\theta_{BW}^{\prime }/\cos\theta$, where $\theta_{BW}^{\prime }$ is the beam-width of the side-looking beam, and substitute it into Equations (1)–(3). It can be obtained that BW1=BW2=BW3 and then the Doppler bandwidth of three beams is the same. Therefore, the three beam images have the same azimuth resolutions. 

The distance between AB is

(4) $$L_{AB}=R_{S}\tan\theta$$

The distance between the BC and the distance between the AB are the same. The time difference between the forward-looking beam and the side-looking beam is

(5) $$\Delta t=L_{AB}/v$$

The repetition frequency of the transmitted pulse is PRF and the data are received in the strip mode. For a same target, it is located at different azimuth sampling units, and the difference of azimuth sampling units between different beams is

(6) $$\Delta nan=\Delta t\cdot PRF=L_{AB}\cdot PRF/v$$

The data of each beam are processed independently to obtain images. When fusing the images from different beams, it is necessary to ensure the matching of the position of the target. According to (6), the forward-looking image is moved back by 2Δnan azimuth sampling units, and the side-looking image is moved back by Δnan azimuth sampling units. The images obtained from the three beams are fused in backward-looking image.

## 3. Multi-Angle SAR Imaging Method Based on a Unified Coordinate

### 3.1. Problems of Multi-Angle SAR Registrations and Fusion

The fusion objects of current SAR images are various remote sensing images, including the fusion of infrared images and SAR images, the fusion of optical images and SAR images, and the fusion of SAR images. Most current SAR image registrations are performed in the image domain. A heterogeneous-SAR image registration method by normalized cross correlation is proposed in [9]. Frost filtering is implemented on the SAR image and then the Gaussian gradient images of SAR image is used to form two Gabor characteristic matrixes, and then the normalized cross correlation matching is implemented on the two characteristic matrixes to achieve the registration of the image. The edge features of the target and the feature points can be extracted from the SAR image [10,11,12,13], and the SAR image registration is performed by the information. A new method is proposed in [10] to detect stable features by intersecting Coherent Scatters. The stable features are used to achieve the coarse registration and the Powell algorithm is used for precise registration. A new method using boundary features of images to achieve SAR image registration is proposed in [12]. A globalized boundary detection algorithm is used for feature extraction and the coherence point drift algorithm is used to match the boundaries. A method for non-homologous SAR image registration is proposed in [14]. The method utilizes multi-look technology to multi resolution images, then uses the coherent phase to deal with multi resolution images, respectively, getting the registration point and achieving image registration.

### 3.2. Improved RMA Algorithm

RMA [15,16,17,18,19,20,21] achieves SAR imaging in the wave number domain. In spite of the squint angle value, it can perfectly focus the whole scene without using any approximate conditions. The range cell migration compensation, secondary range compression and azimuth compression are achieved by Stolt interpolation [15]. In principle, it is the optimum algorithm for SAR imaging [16]. However, the Stolt interpolation needs huge computation. Since the multi-angle SAR imaging system adopts the method of multi-beamforming, the beam squint angle is more than 20°, and the RMA algorithm can process the data of the squint SAR. It can focus the whole scene by interpolation. For 20° squint, the general interpolation formula has low spectrum utilization (Section 3.3. for details.), and the improved RMA algorithm is used to improve the spectrum utilization.

**Figure 4:**
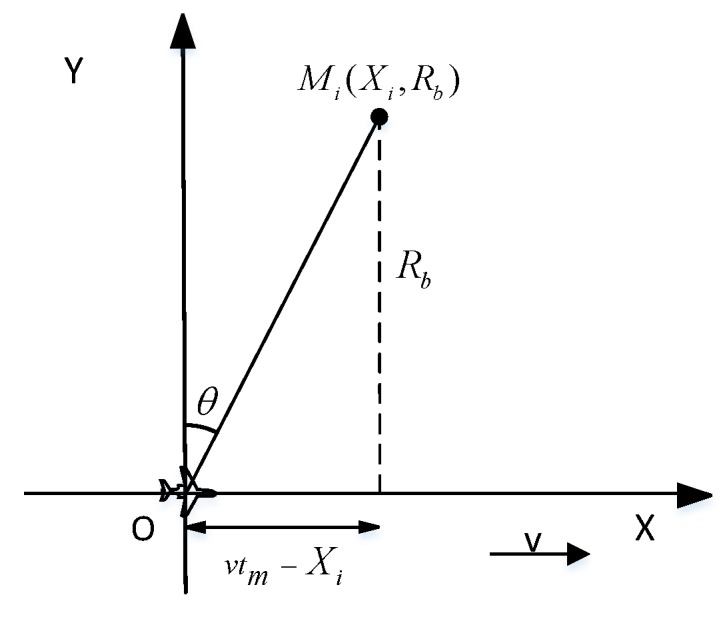
Single beam signal model.

To illustrate the derivation process of the echo signal, the imaging relationship at point A in Figure 3 is drawn separately, as shown in Figure 4. $M_{i}(X_{i},R_{b})$ is one point in the scenario and $R_{b}$ is the closest distance from the point target to the aircraft trajectory. The distance from the aircraft to the point target can be expressed as:

(7) $$R(t_{m})=\sqrt{R_{b}^{2}+{\left(vt_{m}-X_{i}\right)}^{2}}$$

where $t_{m}$ is the azimuth slow-time. Assuming that the transmitted signal is a LFM signal, the received baseband echo signal is [17]:

(8) $$s_{0}(t_{r},t_{m})=A_{0}\omega_{r}\left(t_{r}-2\frac{R\left(t_{m}\right)}{c}\right)\omega_{a}\left(t_{m}-t_{mc}\right)\exp\left\{-j4\pi f_{c}\frac{R\left(t_{m}\right)}{c}\right\}\exp\left\{j\pi \gamma {\left(t_{r}-\frac{2R\left(t_{m}\right)}{c}\right)}^{2}\right\}$$

where $A_{0}$ is the amplitude of the signal, $\omega_{r}(\cdot )$ is the range envelope, $t_{r}$ is the range fast time, $\omega_{a}(\cdot )$ is the azimuth envelope, $t_{mc}$ is the center of synthetic aperture time, $f_{c}$ is the center frequency of the transmitted signal, and γ is the chirp rate of the chirp signal. A two-dimensional FFT is applied to the echo signal, and the two-dimensional frequency domain expression can be obtained:

(9) $$S_{2DF}\left(f_{r},f_{a}\right)=A_{1}W_{r}\left(f_{r}\right)W_{a}\left(f_{a}-f_{ac}\right)\exp\left\{j\theta_{2DF}\left(f_{r},f_{a}\right)\right\}$$

where

(10) $$\theta_{2DF}(f_{r},f_{a})=-\frac{4\pi R_{b}(f_{c}+f_{r})}{c}\sqrt{1-\frac{{\left(cf_{a}\right)}^{2}}{4{\left(f_{c}+f_{r}\right)}^{2}v^{2}}}-\frac{\pi f_{r}^{2}}{\gamma }-2\pi f_{a}\frac{X_{i}}{v}$$

$W_{a}\left(f_{a}\right)=w_{a}\left(\frac{-cR_{0}f_{a}}{2\left(f_{c}+f_{r}\right)v^{2}\sqrt{1-\frac{c^{2}f_{a}^{2}}{4v^{2}{\left(f_{c}+f_{r}\right)}^{2}}}}\right)$ is the envelope of the azimuth spectrum, and $W_{r}\left(f_{r}\right)=\omega_{r}(\frac{f_{r}}{\gamma })$ is the envelope of the range spectrum.

Pulse compression needs to eliminate the quadratic term of $f_{r}$ in Equation (10), and a matched filter can be constructed in frequency:

(11) $$H_{r}(f_{r})=\exp(j\frac{\pi f_{r}^{2}}{\gamma })$$

After multiplication of Equation (9) and Equation (11) to complete pulse compression, the phase after pulse compression is:

(12) $$\theta (f_{r},f_{a})=-\frac{4\pi R_{b}(f_{c}+f_{r})}{c}\sqrt{1-\frac{{\left(cf_{a}\right)}^{2}}{4{\left(f_{c}+f_{r}\right)}^{2}v^{2}}}-2\pi f_{a}\frac{X_{i}}{v}$$

Let $k_{r}=\frac{4\pi (f_{r}+f_{c})}{c}$, $k_{x}=\frac{2\pi f_{a}}{v}$, Formula (12) is rewritten as:

(13) $$\theta \left(k_{r},k_{x}\right)=-R_{b}\sqrt{k_{r}^{2}-k_{x}^{2}}-k_{x}X_{i}$$

Since the signal processing of the RMA algorithm is performed in the two-dimensional frequency domain, and $R_{b}$ represents the time domain, the phase compensation cannot handle the change along the range direction. At this time, a reference range is first selected, and the phase at the reference distance is compensated. Generally, the reference range is set at the center of the scenario. At this time, the matched function of consistent compression is:

(14) $$H_{COMP}\left(k_{r},k_{x}\right)=jR_{S}\sqrt{k_{r}^{2}-k_{x}^{2}}+jk_{x}R_{s}\tan\theta$$

where, $R_{s}$ is the closest distance from the center point of the scenario to the aircraft trajectory. After consistent compression, the point at the center of the scenario is completely focused, and the residual phase at the other range is:

(15) $$\theta_{RFM}\left(k_{r},k_{x}\right)=-\left(R_{b}-R_{S}\right)\sqrt{k_{r}^{2}-k_{x}^{2}}-k_{x}(X_{i}-R_{s}\tan\theta )$$

**Figure 5:**
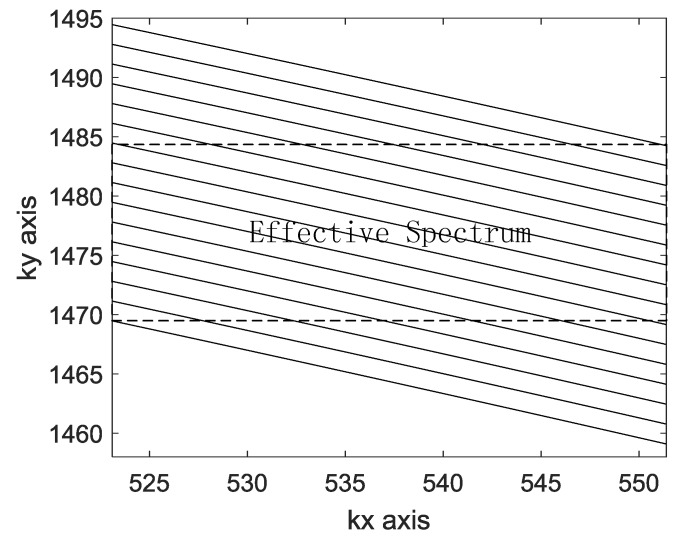
Spectrum of traditional interpolation.

The RMA algorithm performs range cell migration compensation, secondary range compression and azimuth compression by interpolation $k_{y}=\sqrt{k_{r}^{2}-k_{x}^{2}}$ [1,17]. For 20° squint, the two-dimensional spectrum is distorted, and it needs to extract a rectangular aperture of data adequately in such 2-D support [18], as shown in Figure 5; it needs to discard part of the spectrum due to the squint angle, which reduces the energy of targets after imaging. For each of the determined $k_{r}$, the variation of $k_{y}$ with $k_{x}$ is shown by the arc in Figure 5. 

The improved interpolation uses the tangent of each arc instead of the traditional $k_{y}$, and corrects the distorted spectrum. Therefore, the method can effectively improve the utilization of the spectrum in squint SAR. The improved interpolation is:

(16) $$k_{y}=\sqrt{k_{r}^{2}-k_{x}^{2}}-\left[\sqrt{k_{rc}^{2}-k_{xc}^{2}}-\frac{k_{xc}}{\sqrt{k_{rc}^{2}-k_{xc}^{2}}}\left(k_{x}-k_{xc}\right)\right]$$

where $k_{rc}=\frac{4\pi f_{c}}{c}$, $k_{xc}=\frac{2\pi f_{ac}}{c}$ and $f_{ac}=\frac{2v\sin\theta }{\lambda }$ is Doppler center. The residual phase after interpolation is

(17) $$\theta_{STOLT}\left(k_{y},k_{x}\right)=-\left(R_{b}-R_{S}\right)\left[k_{y}+\left(\sqrt{k_{rc}^{2}-k_{xc}^{2}}-\frac{k_{xc}}{\sqrt{k_{rc}^{2}-k_{xc}^{2}}}\left(k_{x}-k_{xc}\right)\right)\right]-k_{x}(X_{i}-R_{s}\tan\theta )$$

Since the interpolation introduces a linear phase that varies with range, it is necessary to compensate for the introduced linear phase in the Range–Doppler domain. After IFFT along the range, the following is obtained:

(18) $$\begin{array}{cc} s_{RD}(Y,k_{x}) & =A_{2}\sin c\left(\frac{B_{ky}}{2\pi }Y\right)W_{a}\left(\frac{vk_{x}}{2\pi }\right)\exp\left\{-jk_{x}(X_{i}-R_{s}\tan\theta )\right\}\\ & \cdot \exp\left\{-j\left(R_{b}-R_{S}\right)\left(\sqrt{k_{rc}^{2}-k_{xc}^{2}}-\frac{k_{xc}}{\sqrt{k_{rc}^{2}-k_{xc}^{2}}}\left(k_{x}-k_{xc}\right)\right)\right\} \end{array}$$

where $B_{ky}$ is the bandwidth of $k_{y}$, $Y=R_{b}-R_{S}$, and the second phase in Equation (18) needs to be compensated along azimuth, and the azimuth compensation function is:

(19) $$H_{AZIMUTH}\left(R_{b},k_{x}\right)=\exp\left\{j\left(R_{b}-R_{S}\right)\left(\sqrt{k_{rc}^{2}-k_{xc}^{2}}-\frac{k_{xc}}{\sqrt{k_{rc}^{2}-k_{xc}^{2}}}\left(k_{x}-k_{xc}\right)\right)\right\}$$

Multiply Equation (18) and Equation (19) and perform IFFT along azimuth to obtain:

(20) $$s_{RX}(Y,X_{i})=A_{3}\sin c\left(\frac{B_{ky}}{2\pi }Y\right)\sin c\left(\frac{B_{kx}}{2\pi }(X_{i}-R_{s}\tan\theta )\right)$$

where $B_{kx}$ is the bandwidth of $k_{x}$. The point target $M_{i}(X_{i},R_{b})$ is focused at $\left(X_{i}-R_{s}\tan\theta ,R_{b}-R_{S}\right)$ in the time domain.

**Figure 6:**
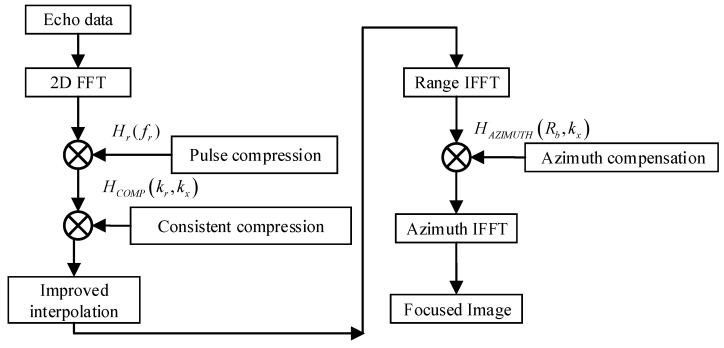
Multi-angle SAR algorithm flow chart.

The algorithm processing flow is shown in Figure 6:

### 3.3. Application and Consideration

**Figure 7:**
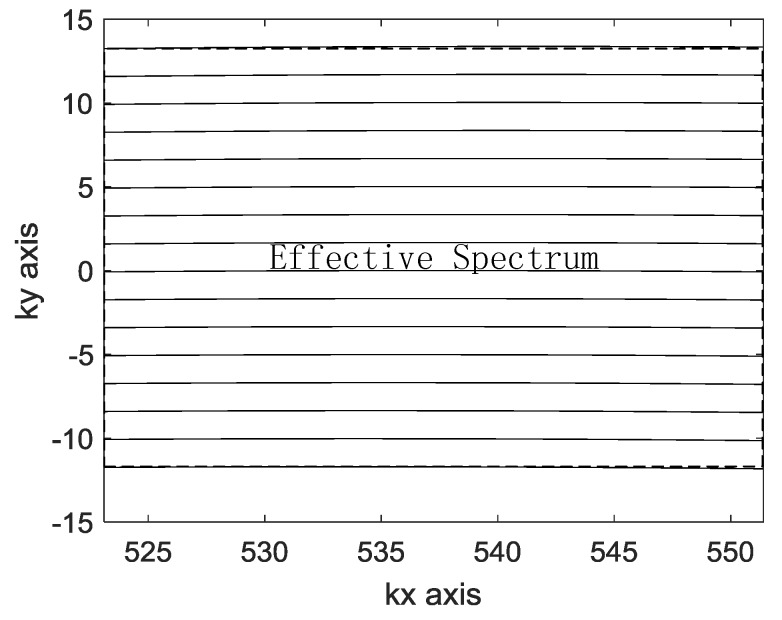
Spectrum of proposed interpolation.

For many artificial objects, the SAR image is greatly affected by the azimuth angle. Through multi-angle image fusion, we can obtain more detailed information about the target, which improves the target detection and recognition ability of SAR images. SAR image matching fusion can be achieved quickly by imaging in a unified coordinate system. In order to maximize the use of the spectrum, it is necessary to make the interpolated spectrum as rectangular as possible. After interpolation, the original coordinate axis $k_{r}$ is replaced by the new coordinate axis $k_{y}$. Figure 7 is the bandwidth of the spectrum after interpolation, and the effective spectrum is the part within the dashed box. Figure 5 shows the spectrum of the traditional interpolation method, and the spectrum is approximated as a character quadrilateral. The effective spectrum is significantly smaller than the spectrum obtained by the method of this paper.

**Figure 8:**
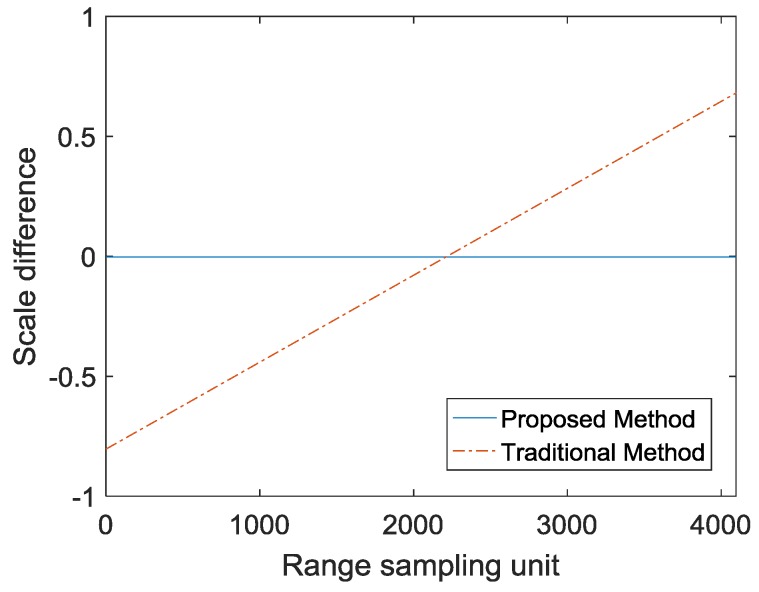
Scale difference of two methods.

**Figure 9:**
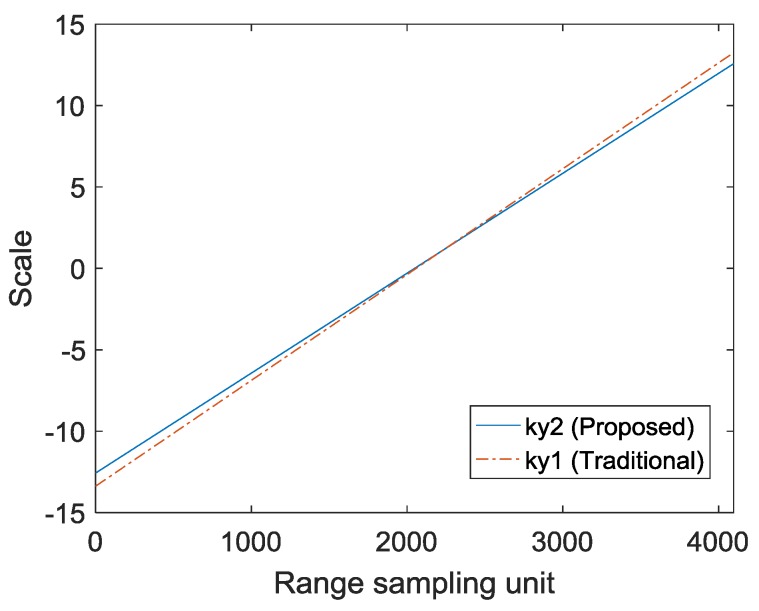
Comparison of two sampling methods.

For accurate matching, images need to have a uniform scale. The bandwidth of $k_{y}$ represents the range bandwidth after interpolation. In order to have the same range resolution of multiple-angle images in the time domain, the bandwidth of $k_{y}$ is required to be the same. The traditional method is to intercept the largest rectangle in the interpolated spectrum, as shown in Figure 5. The traditional method is used to determine $k_{y1}$. Let $k_{rL}=\min\left(kr\right)$, $k_{rH}=\max\left(kr\right)$, $k_{yL}=\max\left(\sqrt{k_{rL}^{2}-k_{x}^{2}}\right)$, $k_{yH}=\min\left(\sqrt{k_{rH}^{2}-k_{x}^{2}}\right)$ and N is the number of range sampling units, and then $k_{y1}\left(i\right)=k_{yL}+\left(i-1\right)\left(k_{yH}-k_{yL}\right)/N,\;i=1,2,\cdots ,N$. The result of $k_{y1}-(k_{r}-k_{rc})$ is shown in Figure 8. The slope greater than 0 represents the bandwidth of $k_{y1}$ is greater than the bandwidth of $k_{r}$. In the images of different views, the bandwidth of $k_{y1}$ is inconsistent and there is a slight change in the range resolution of the time domain. In general SAR imaging applications, it can be ignored. However, the change in the range resolution will lead to inaccurate matching and affect the quality of the fusion in image matching. In the proposed method, in order to unify the bandwidth of $k_{y}$ in different view images, the center value of $k_{y}$ is first determined, and then the bandwidth of $k_{y}$ is determined according to the bandwidth of the $k_{r}$. The proposed method is used to determine $k_{y2}$ and $k_{y2}\left(i\right)=k_{rc}+\left(i-N/2\right)\left(k_{rH}-k_{rL}\right)/N,i=1,2,\cdots ,N$. As shown in Figure 9, the bandwidth of $k_{y2}$ is smaller than the bandwidth of $k_{y1}$, which means that the proposed method discards a small portion of the spectrum. The result of $k_{y2}-(k_{r}-k_{rc})$ is shown in Figure 8. The slope is 0, which represents the bandwidth of $k_{y2}$ is the same as the bandwidth of $k_{r}$. In the images of different views, the bandwidth of $k_{y2}$ is consistent, and different images have the same range resolution in the time domain. The advantage of the scale uniformity is obvious in image matching.

It is difficult for the aircraft to maintain an ideal state due to factors such as airflow during flight. Therefore, motion compensation is required in data processing. In the mode of multi-flight acquisition for imaging data, the motion compensation of each SAR image is different because of the different motion errors of each flight, which brings difficulties to image matching. When multi-angle SAR data are taken by this system, data of each angle have the same motion error, and the data of multiple angles can be compensated by the motion error of a single view, simplifying the compensation process. It is also possible to jointly perform motion compensation through multiple viewing angles to improve compensation accuracy.

## 4. Experimental Simulation, Measured Data

### 4.1. Experimental Simulation

**Figure 10:**
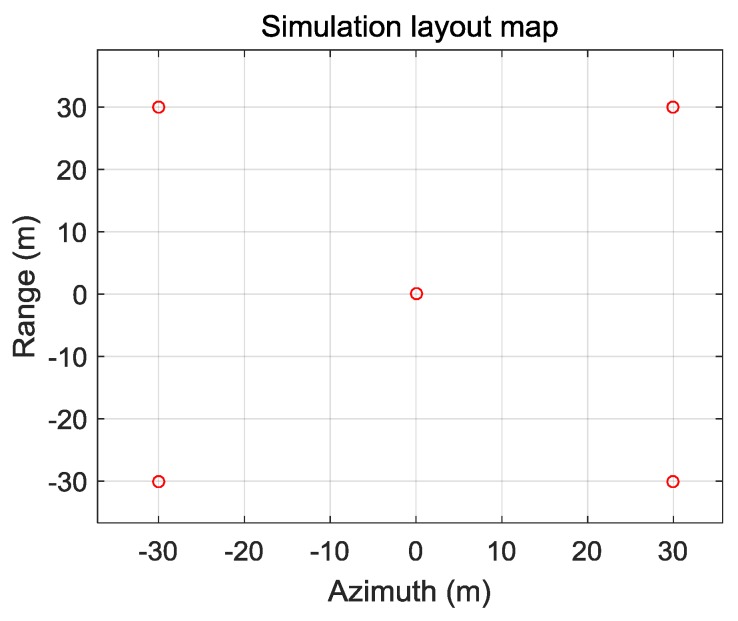
Simulation layout map.

In order to verify the validity of the algorithm, the simulation data are used for explanation. The simulation resolution is 0.3 m × 0.3 m, the wavelength is 3 cm, the center frequency is10 GHz, the signal bandwidth is 500 MHz, the range sampling rate is 600 MHz, the pulse width is 3.5 µs, the speed of aircraft is 100 m/s, the antenna aperture is 0.6 m, and the pulse repetition frequency is 450 Hz. The closest distance from the center of the scenario to the aircraft route is 30 km. Three beams are used with a beam spacing of 20° and the beam width is 2.86°. There are five points in the scene, and the simulation scenario layout is shown in Figure 10. The center point target is located at (0, 0), and the remaining four points are located at (±30, ±30).

**Figure 11:**
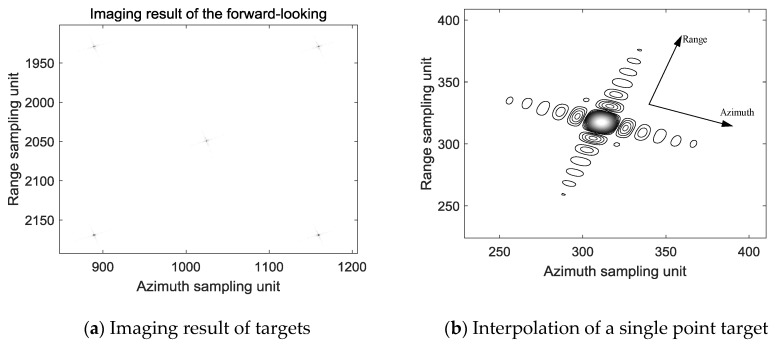

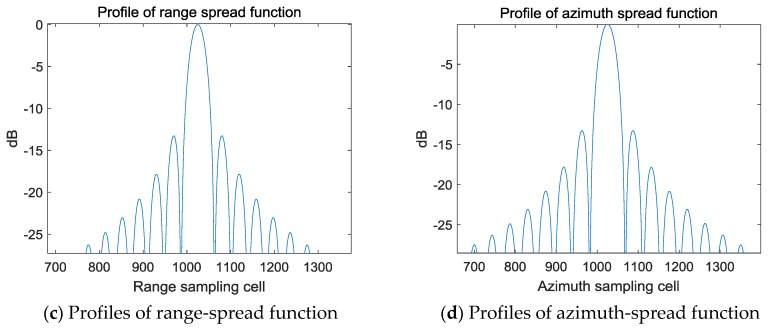
Imaging result of the forward-looking beam.

The squint angle of the forward-looking beam is 20°, and the scenario image processed by the above imaging algorithm is shown in Figure 11a, the position of the center point target is (1025, 2050), and the positions of the other four points are (1025 ± 135, 2050 ± 120). The azimuth sampling rate is 1.35 times of the azimuth bandwidth, so the distance between the center point target and the rest of the point target in the azimuth direction is 135/1.35 × 0.3 = 30 m, which is consistent with the scenario layout; The range sampling rate is 1.2 times of the bandwidth, and the distance between the center point target and the rest of the point target in the range direction is 120/1.2 × 0.3 = 30 m, which is consistent with the scenario layout. Figure 11b is a result of interpolation of the point (1025 − 135, 2050 − 120) in Figure 11a. It can be seen that the point target in forward-looking beam is well focused. The profiles of range and azimuth-spread function of the target are presented in Figure 11c,d. The peak sidelobe ratio (PLSR) along the range direction shown in Figure 11b is −13.2242. The integral sidelobe ratio (ISLR) along the range direction is −9.8468. The PLSR along the azimuth direction shown in Figure 11b is −13.2611. The ISLR along the azimuth direction is −9.8963.

**Figure 12:**
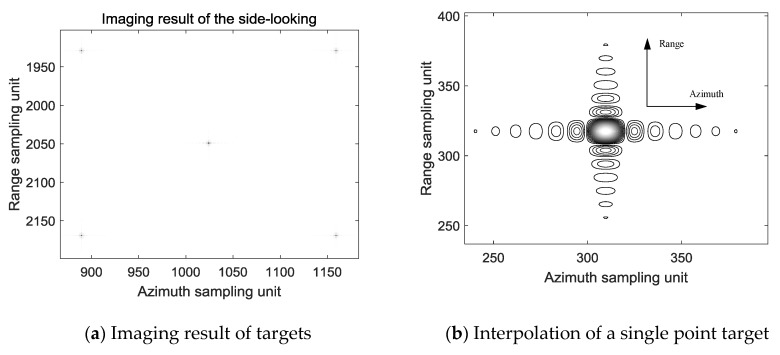

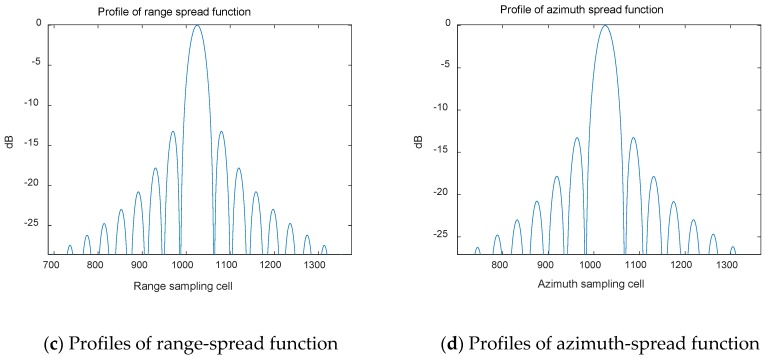
Imaging result of the side-looking beam.

The scenario image of the side-looking beam processed by the above imaging algorithm is shown in Figure 12a, the position of the center point target is (1025, 2050), and the positions of the remaining four points are (1025 ± 135, 2050 ± 120). The distance between the center point target and the rest of the point target in the azimuth direction is 135/1.35 × 0.3 = 30 m, and the distance between the center point target and the rest of the point target in the range direction is 120/1.2 × 0.3 = 30 m, which is consistent with the scenario layout. Figure 12b is a result of interpolation of the point (1025 − 135, 2050 − 120) in Figure 12a. It can be seen that the point target in side-looking beam is well focused. The profiles of range and azimuth-spread function of the target are presented in Figure 12c,d. The PLSR along the range direction shown in Figure 12b is −13.2231. The ISLR along the range direction is −9.8464. The PLSR along the azimuth direction shown in Figure 12b is −13.2602. The ISLR along the azimuth direction is −9.8962.

**Figure 13:**
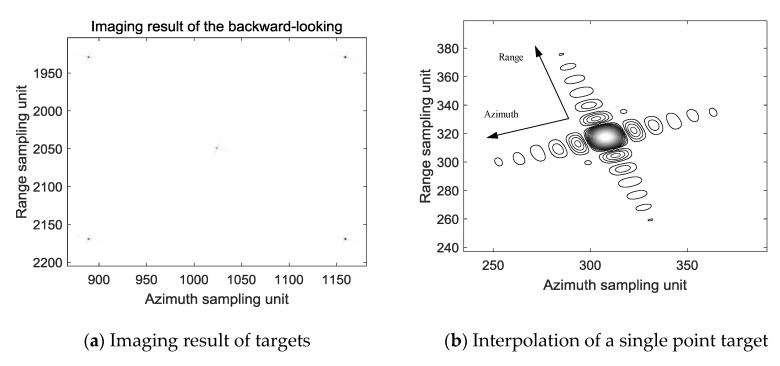

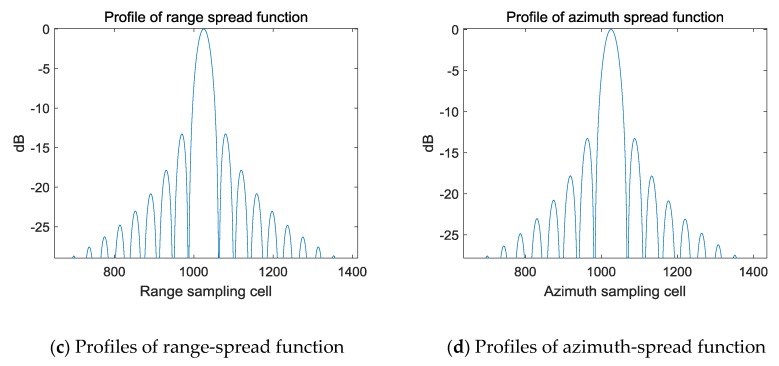
Imaging result of the backward-looking beam.

The scenario image processed by the above imaging algorithm for the backward-looking beam is shown in Figure 13a. the position of the center point target is (1025, 2050), and the positions of the remaining four points are (1025 ± 135, 2050 ± 120). The distance between the center point target and the rest of the point target in the azimuth direction is 135/1.35 × 0.3 = 30 m, and the distance between the center point target and the rest of the point target in the range direction is 120/1.2 × 0.3 = 30 m, which is consistent with the scenario layout. Figure 13b is a result of interpolation of the point (1025 − 135, 2050 − 120) in Figure 12a. It can be seen that the point target in backward-looking beam is well focused. The profiles of range and azimuth-spread function of the target are presented in Figure 13c,d. The PLSR along the range direction shown in Figure 13b is −13.2299. The ISLR along the range direction is −9.8458. The PLSR along the azimuth direction shown in Figure 13b is −13.2536. The ISLR along the azimuth direction is −9.8859.

In each beam, the absolute position and relative position of the point target are not changed and matched with the ground point, so the imaging of the same point target on the ground by different beams only has the difference in azimuth time. According to the time difference represented by Formula (5) or the azimuth point difference represented by Formula (6), the image fusion of multi-view SAR can be completed by delaying the forward-looking beam imaging result by 2Δt and delaying the side-looking beam imaging result by Δt, and then superimposing them into the backward-looking beam imaging result.

**Figure 14:**
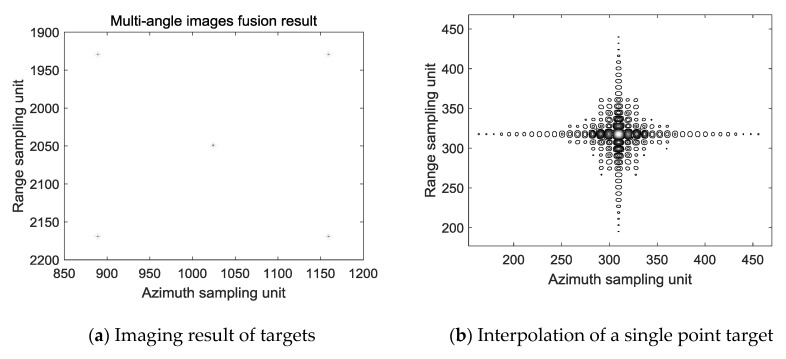

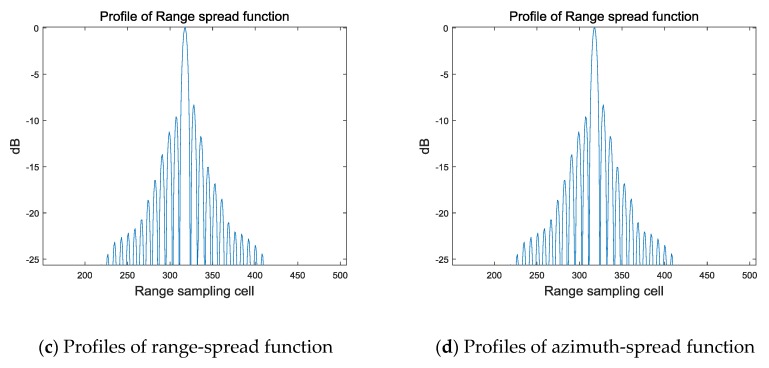
Result after image fusion.

According to the time difference represented by the Formula (5), or the difference in the number of azimuth points represented by the Formula (6), the front-view beam imaging result is delayed by 2Δ*t*, the due side-view imaging result is delayed by Δ*t*, and then image fusion of multi-angle SAR is completed after superimposition on back-view beam. The result of the fusion is shown in Figure 14a. Figure 14b is a result of interpolation of the point in Figure 14a. It can be seen from Figure 14a,b that the imaging and fusion of images can be completed in a uniform coordinate system within a viewing angle range of −20° to 20°. The Range PSLR is −8.31 and the azimuth PSLR is −6.37.

### 4.2. Measured Data

In order to validate the effectiveness of the proposed algorithm, the large-angle spotlight SAR measured data are processed using the proposed algorithm. The large-angle spotlight SAR measured data contains information about multiple perspectives of the target. After dividing the data into two parts according to the two viewpoints of forward-looking and backward-looking, the fusion image of multi-angle SAR is obtained by using the algorithm proposed in this paper. The parameters of the system are shown in Table 1.

**Figure 15:**
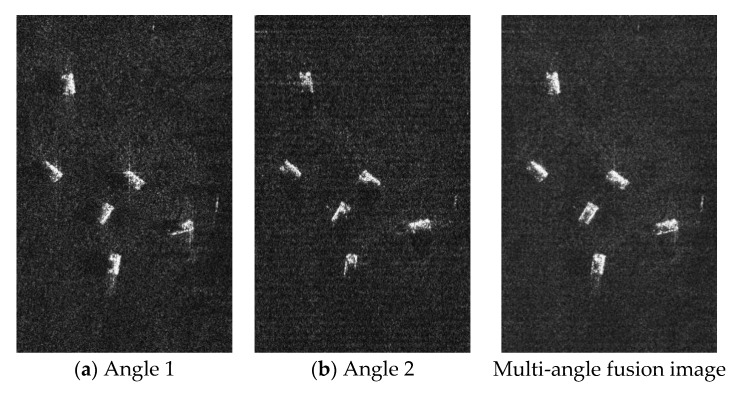
Image fusion results of proposed method.

Figure 15a,b are images of six vehicles with forward-looking and backward-looking views. It can be seen that the target information obtained is not complete because of sheltering of the single-view target. Figure 15c is obtained through the image fusion of two angles of view. From which, complete geometric features of the target can be seen clearly. The information entropy is used to evaluate the effects of image fusion. Information entropy in Figure 15a,b are 6.1819 and 6.1046, and information entropy in Figure 15c is 6.6635. The information entropy in the image increases after fusion. This means the fused image contains more information about the targets.

**Figure 16:**
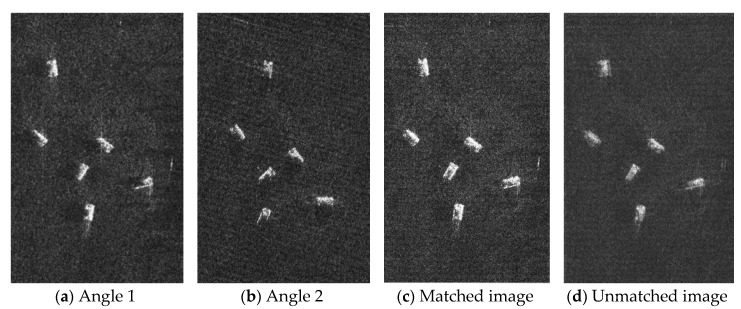
Image fusion results of Range–Doppler algorithm.

Figure 16 shows the image fusion results of Range–Doppler algorithm. Different from the proposed method, the result of angle 2 has a deformation, and the image registration needs to be performed after the image is corrected. When the images are fully registered, the images can be well fused as shown in Figure 16c. When the image is not fully registered, part of the target information will be lost as shown in Figure 16d.

Compared with the traditional method, the method proposed in this paper does not require additional image registration, which simplifies the process of image fusion. It also avoids the effects of mismatch between images. However, RMA requires interpolation and is computationally intensive, which can cause real-time processing difficulties.

**Figure 17:**
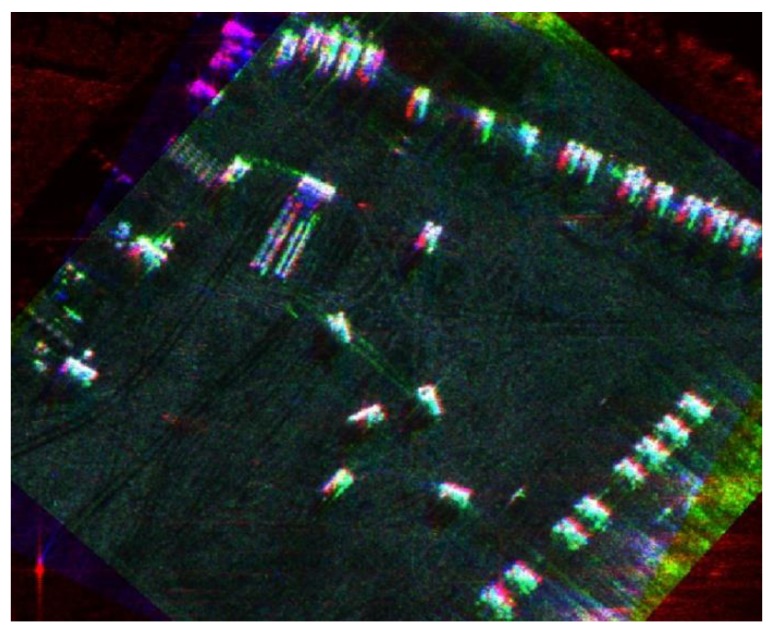
A multi-angle fusion result of a large scenario area.

Figure 17 shows a multi-angle fusion result of two views in a large scenario area, in which red represent the components of forward-looking view and green represent the components of backward-looking view. The background is spotlight SAR image, and the segmented portion is forward-looking and backward-looking images.

**Figure 18:**
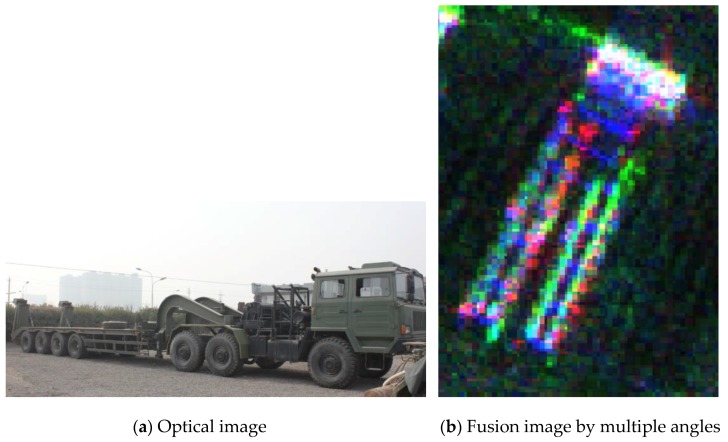
Optical image and fusion image of a vehicle.

Figure 18 is an optical picture and enlarged fusion result of the transport vehicle of Figure 18. Different colors represent components of different views. In a single view image, the occluded portion can be supplemented by another view. The geometric characteristics of the transport vehicle are relatively complete, which is beneficial to the identification of the target.

## 5. Conclusions

A multi-angle SAR imaging system is proposed in this paper using multi-beamforming. When using an ultrahigh speed platform, the main issue is an increase in Doppler bandwidth in the signal. As a result, it is difficult to separate signals of multiple beams in the frequency domain. Therefore, this paper separates the multi-beam signal in the time domain using three groups of feeders. In order to achieve accurate matching of multi-view SAR images, an improved RMA in a unified coordinate is proposed. SAR data from different view angles is imaged in a uniform coordinate system. The resolution between images is the same, and the image is not deformed and scaled. There is only a time delay relationship between images of different view angle. Therefore, image fusion does not require additional registration. Multi-angle images can be quickly and accurately fused.

## Figures and Tables

**Table 1:** Parameters of the system.

Parameters	Value
Velocity	102 m/s
Frequency band	9.6 GHz
Bandwidth	600 MHz
PRF	312 Hz
Angle range	−30°–30°
Reference range	34 km
